# Desflurane anaesthesia in myotonic dystrophy

**DOI:** 10.4103/0019-5049.76599

**Published:** 2011

**Authors:** Ranju Gandhi, Anil Kumar Jain, Jayashree Sood

**Affiliations:** Department of Anaesthesiology, Pain and Perioperative Medicine, Sir Ganga Ram Hospital, New Delhi, India

**Keywords:** Desflurane anaesthesia, myotonic dystrophy, laparoscopic cholecystectomy

## Abstract

Myotonic dystrophy (MD) is a rare genetic disorder with multisystem involvement characterised by myotonia and progressive muscle weakness and wasting. These patients pose significant challenges to the anaesthesiologist in view of the muscular and extramuscular involvement and sensitivity to anaesthetic drugs. The literature is replete with reports of postanaesthetic respiratory and cardiovascular complications in these patients. But an ideal anaesthetic technique in MD patients remains to be determined. Rapid recovery is desirable to reduce postoperative respiratory complications. Though there are a few case reports of maintenance of anaesthesia with isoflurane and sevoflurane, there are scanty reports of use of desflurane in these patients. We present successful management of a patient with MD for laparoscopic cholecystectomy using a carefully titrated desflurane-based anaesthesia and discuss the perioperative considerations.

## INTRODUCTION

Myotonias are a group of hereditary degenerative diseases characterized by myotonia (persistent contracture and delayed relaxation of the skeletal muscle after voluntary contracture or stimulation of the muscle). The most common myotonic dystrophy (MD) is type 1 (Steinert disease), others being proximal myotonic myopathy, myotonic dystrophy type 2 and proximal myotonic dystrophy. Type 1 MD is inherited in an autosomal dominant pattern with variable expression in both clinical severity and age at onset caused by an abnormal expansion of the CTG trinucleotide repeat in the protein kinase gene on chromosome 19q13.3. Symptoms typically appear in the second or third decade of life, though a congenital form also exists. Numerous case reports of postanaesthetic myotonic crisis, prolonged apnea, delayed recovery, arrhythmias, need for prolonged ventilatory support, postoperative aspiration, hypoventilation, respiratory failure and even deaths in these patients underscore the seriousness of the disorder.[1–3] Depolarizing muscle relaxants are hazardous while the response to non-depolarizing muscle relaxants is reported to be normal.[4] There has been reluctance to use volatile anaesthetics for fear of malignant hyperthermia (MH) and their cardiorespiratory depressant effects. The recent literature does not support an association between MH and MD.[5] The use of isoflurane and sevoflurane but not of desflurane has been described in patients with MD.[6]

## CASE REPORT

A 33 year old male patient, weighing 60 kg, 165 cm tall and a known case of MD type 1 confirmed by a genetic study, presented to our hospital with symptomatic gall stone disease. He was planned for laparoscopic cholecystectomy. He had restricted physical activities. The past history revealed seizures following cerebral cyst excision 18 years back under general anaesthesia and ventilatory support postoperatively. Seizures were controlled on tablet phenytoin which was stopped 6 months back. On examination, he had frontal balding, bilateral ptosis, an expressionless face, a large mandible, bucked teeth, dorsal kyphosis, slurred speech and slow gait. Chest auscultation revealed bilateral conducted sounds. The cardiovascular system, haematological and biochemical investigations and chest X-ray were within normal limits. Pulmonary function tests revealed moderate–severe restrictive disease. Electrocardiogram (ECG) showed a right axis deviation but echocardiogram was normal. Informed consent was taken and need for care in postoperative intensive care unit explained to the family.

The patient was made to fast overnight and was not premedicated. The ambient temperature of the operating suite was raised before wheeling the patient inside. Standard and neuromuscular monitors were applied. Anaesthesia was induced with fentanyl 30 µg; propofol 50 mg was given slowly and atracurium 15 mg in a head-up position to avoid aspiration. At 190 s there was single twitch suppression of 90%. He required an additional 5 mg of atracurium and the trachea was intubated after ventilating for another 30 s. Intraoperative monitoring included 5-lead ECG, noninvasive blood pressure (NIBP), pulse oximeter (SpO_2_), end tidal carbon dioxide (EtCO_2_), temperature, MAC (minimum alveolar concentration) and train of four (TOF) with neuromuscular monitor. Anaesthesia was maintained with 50% nitrous oxide and 4–6% end-tidal desflurane in oxygen. Warmed intravenous (I.V) fluids and convective forced air warmer ensured normothermia. Haemodynamics remained stable throughout the surgery lasting 90 min. At the end of the surgery, 20 ml 0.25% bupivacaine was given intraperitoneally and port sites were infiltrated as well. Neuromuscular blockade was reversed when the TOF count was 4 with 0.25 mg glycopyrrolate and 1.25 mg neostigmine. The trachea was extubated once the TOF ratio was 90%, respiratory efforts were adequate and the patient was following commands, about 8 min after switching off desflurane. He was shifted to the postoperative recovery room, kept warm, given oxygen, and analgesia was provided with diclofenac sodium 75 mg. He was kept in intensive care unit for 24 h and had an uneventful course.

## DISCUSSION

Patients with MD have progressive weakness and wasting of muscles of mastication, neck, pharynx and distal limbs. Although skeletal muscles are affected the most, cardiac (conduction system) and smooth muscles (gastrointestinal tract) can also be affected. Extramuscular manifestations include cataract, premature balding, mental retardation, sleep apnea, diabetes mellitus and thyroid, adrenal and gonadal dysfunction. Heart block, mitral valve prolapse, cardiomyopathy, cardiac failure and sudden death are also reported. These patients may have restrictive lung disease, mild arterial hypoxemia and diminished ventilatory responses to hypercapnia and hypoxia. The pulmonary complications are the result of hypotonia, aspiration and central hypoventilation which can be exacerbated after anaesthesia.

Factors contributing to increased complications are emergency surgery, upper abdominal surgery, preoperative significant respiratory cripple, perioperative morphine, use of a muscle relaxant without reversal, long surgery and an undiagnosed case.[1–3] Myotonic crisis can be precipitated intraoperatively by succinylcholine, etomidate, methohexital and neostigmine and physical factors like hypothermia, electrocautery and surgical stimulation. A generalized and/or localized contracture (myotonia) should be prevented by prewarming the operation theater (OT) and IV fluids, avoidance of succinylcholine and shivering and gentle handling of muscles during surgery. Medications for treatment of myotonia, mexilitine and phenytoin, should be readily available. It is prudent to do neuromuscular monitoring and avoid residual paralysis.

We could successfully manage our patient using carefully titrated doses of atracurium, fentanyl and desflurane. Propofol has been used in patients with myotonia with variable responses, including prolonged recovery, altered dose-response curves, precipitation of myotonias and uneventful administration.[7,8] Considering this we used propofol for induction only and not for maintenance of anaesthesia. As our patient had absence of cardiovascular system involvement, we used desflurane for the maintenance of anaesthesia and it provided good control over anaesthetic depth as well as haemodynamics. The requirement of atracurium was less in our patient and was guided by neuromuscular monitoring. Intraoperative bupivacaine reduced the need for opioids in the postoperative period which can cause paralytic ileus. As per our knowledge, the use of desflurane in MD patients has not been reported. Desflurane with its low solubility in blood and tissues can be an ideal agent for these patients in view of its better recovery profile than any other agent. Its cessation at the end of surgery leads to rapid return of airway reflexes and faster awakening.

Thoracic epidural anaesthesia (TEA) has been used in a MD patient for laparoscopic cholecystectomy.[9] But we did not consider that option as our patient had dorsal kyphosis. Moreover, laparoscopic surgery under TEA can lead to shoulder pain, nausea, vomiting and patient discomfort. It has an attendant risk of a high motor block with cardiorespiratory compromise which can be detrimental in patients with MD. Others have used sevoflurane without relaxants, PFK (Propofol, fentanyl and ketamine combination), dexmedetomedine, total intravenous anaesthesia with propofol, cisatracurium and remifentanil and target controlled infusion of propofol in these patients.[6,10–12]

To conclude, successful anaesthetic management of MD includes a thorough preoperative assessment, selection of short-acting anaesthetic drugs like desflurane to ensure a faster recovery with no residual respiratory depressant effects, avoidance of triggers of myotonic crisis and close monitoring postoperatively for at least 24 h for prompt management of complications.
